# Fluoride release by restorative materials after applying surface coating agents

**DOI:** 10.6026/97320630019423

**Published:** 2023-04-30

**Authors:** Anamika Parashar, Yogesh Kumar Sharma, Sumit Kumar, Kavitha M, Pallabi Ghosh Saha, Pragya Patel

**Affiliations:** 1 Department of Conservative Dentistry and Endodontics, *DR. B.R. Ambedkar Institute of Dental Sciences and Hospital, Patna, Bihar, India*; 2 Department of Oral and Maxillofacial surgery, Mahatma Gandhi Dental College and Hospital, Jaipur, Rajasthan, India; 3 Department of Public Health Dentistry, King George Medical University, Lucknow, Uttar Pradesh, India; 4 Department of Pediatric and Preventive Dentistry, *Dayananda Sagar College of Dental Sciences*, Bengaluru, Karnataka, India; 5 Department of Conservative Dentistry and Endodontics, *Awadh Dental College* and *Hospital, Jamshedpur*, Jharkhand, India; 6 Department of Conservative Dentistry and Endodontics, People's Dental Academy, Bhopal, Madhya Pradesh, India

**Keywords:** Fluoride, restorative materials, surface coating agents

## Abstract

The prompt use of an enamel surface covering reagent is advised to safeguard the dental restorative substance from mishaps. Therefore, it is of interest to assess the fluoride emitting capabilities of standard GIC, and Zirconomer cement together with surface coverings and without surface coverings. The conventional GIC cement was part of experimental category A while Zirconomer cement was part of category B. For every experimental categories, a set of sixty brass mould prototypes in the form of disc with dimensions: diameter (6±0.1mm) and thickness (2±0.1 mm) were created and subsequently covered with Teflon strip in accordance with the package recommendations. Also, for both experimental categories, such pellets were randomly allocated to three sub-categories of 20 each. For one category petroleum jelly was administered with a cotton bud and then delicately dried under airflow (A3 subcategory and B3 subcategory); for another sub-category G-Coat was laced through a micro-tip dispenser and light treated for twenty seconds (A2 subcategory and B2 subcategory); the rest 20 specimens were left without any coating (A1 subcategory and B1 subcategory). It was observed that in subcategory A1 and A3 there was continuous decline in emission of fluoride ion as the days progressed. However there was an increase in emission of fluoride in A2 subcategory on moving to day 5 from day 1. However, from day 5 onwards decline in fluoride emission was observed in A2 subcategory. It was concluded that both materials studied (GIC and Zirconomer) exhibited fluoride emission whether or not they were surface-coated for protection.

## Background:

Among the most common chronic disorders impacting people is tooth decay. It still poses a serious health threat to the majority of grown-ups and school going children in most developed nations [1]. Prior relatively recent times, tooth decay was uncommon in the majority of developing nations, but this is no longer the case. This is partly because of changes in dietary practices and insufficient exposure to fluoride. In contrary, over the past 20 years, the majority of developed nations have seen a drop in dental cavities as a result of improved public health campaigns, including the successful utilization of FL fluorides, altered habits, and the adoption of novel restorative materials [1, 2]. Recrystallization and the discharge of fluoride ions, which results in anti-cariogenic activity, are desired characteristics of these most recent tooth-colored dental restorations. Glass Ionomer Cement (GIC), that was created by Wilson and Kent in 1972 and is frequently used in contemporary periodontal therapy [2], is one such substance. Because to its advantageous characteristics, including its ability to adhere to dental tissues and exude fluoride across an extended length of time, GIC is the perfect dental restorative material employed in paediatric operational dentistry [3]. However, there are several drawbacks as well, including early moisture susceptibility, poor cosmetics, a slow setting process, weakened mechanical capabilities, and weak good adhesion [3].Zirconia fillers are added to the glass ingredient of GIC to address these drawbacks and strengthen the restoration's structural stability and mechanical attributes. Zirconomer (Zirconia reinforced glass ionomer), which combines endurance, exceptional strength, and persistent fluoride protection, is the perfect posterior dental restorative substance for individuals with a high prevalence of caries [4]. The most well-known substance in the research is the GIC, which is water sensitive (hydrophilic) and contains a significant amount of loosely connected water [5]. The cement constituting ions of calcium, aluminium, and silicate chemical agent will be scrubbed out during the beginning phases of the chemical reaction for setting of cement because of dehydration as well as contamination of the substance present in water as well as saliva. This will cause loss of lustre, a decrease in physical prowess, and increased vulnerability to dissolution. The prompt use of a enamel surface covering reagent is advised to safeguard the dental restorative substance from such accidents [6]. The goal of the current in-vitro experiment was to compare and evaluate the fluoride emission properties of regular GIC, Zirconomer cement, and paired with and without surface coatings.

## Materials and Methods:

The conventional GIC cement was part of experimental category A while Zirconomer cement was part of category B. For every experimental categories, a set of sixty brass mold prototypes in the form of disc with dimensions: diameter (6±0.1mm) and thickhness (2±0.1 mm) were created and subsequently covered with teflon strip in accordance with the package recommendations. To guarantee adequate movement of materials, a glass block was placed over the surface and pressed down with hands. The samples were preserved in their moulds for about ten minutes at 100 percent moisture content and 37°C to prevent dehydration as well as moisture intrusion, and the extra material all around perimeter was cut away using a knife. The pellets' faces were then gently polished using wet silicon carbide paper while submerged in water. Also, for both experimental categories, such pellets were randomly allocated to three sub-categories of 20 each. For one category petroleum jelly was administered with a cotton bud and then delicately dried under airflow (A3 subcategory and B3 subcategory); for another sub-category G-Coat was laced through a micro-tip dispenser and light treated for twenty seconds (A2 subcategory and B2 subcategory); the rest 20 specimens were left without any coating (A1 subcategory and B1 subcategory).The discs were submerged in six separate vacuum sealed plastic containers, each having 50 millilitres of distilled deionized water (experimental liquid), as soon as the crosslinking process was complete [3].They were kept uninterrupted in a incubator adjusted at a temperature of 37°C [3]. Following twenty four hours, the testing solution-containing sample containers were taken out of incubation, and the samples were picked up with sterile, metallic forceps that had been painted with nail polish to protect them from contaminates. They were then shifted to fresh sterile containers comprising fifty millilitres of distilled deionized water and wiped employing absorbing paper sheets for 2 minutes. A 5ml quantity of TISAB II was incorporated into the experimental solution for the purpose of estimating the fluoride emission. In the experimental solution, a fluoride cathode was submerged along with an ion spectrometer, and the information was captured taking ppm as unit of measurement. Fluoride discharge from every experimental solution was evaluated daily for 15 days while the experimental solution was updated every twenty four hours.

## Statistical analysis

Kruskal Wallis and the Mann-Whitney U test were used to statistically analyse the data.

## Results:

The mean emission of fluoride ion in A1 subcategory at day 1 was 2.912 ±0.169ppm. The mean emission of fluoride in A2 subcategory at day 1 was 0.431 ±0.023 ppm. The mean emission of fluoride in A3 subcategory at day 1 was 0.441 ±0.024 ppm. The variations in the values in different subcategory in category A was statistically meaningful (p <0.001) with maximum emission of fluoride ion in subcategory A1 at day 1. The mean emission of fluoride ion in A1 subcategory at day 5 was 0.671 ±0.027 ppm. The mean emission of fluoride in A2 subcategory at day 2 was 0.701 ± 0.027 ppm. The mean emission of fluoride in A3 subcategory at day 5 was 0.161 ± 0.021 ppm. The variations in the values in different subcategory in category A was statistically meaningful (p <0.001) with maximum emission of fluoride ion in subcategory A2 at day 5.The mean emission of fluoride ion in A1 subcategory at day 10 was 0.271 ±0.021ppm. The mean emission of fluoride in A2 subcategory at day 10 was 0.341 ± 0.024 ppm. The mean emission of fluoride in A3 subcategory at day 10 was 0.132 ±0.021 ppm. The variations in the values in different subcategory in category A was statistically meaningful (p <0.001) with maximum emission of fluoride ion in subcategory A2 at day 10.The mean emission of fluoride ion in A1 subcategory at day 15 was 0.231 ±0.027 ppm. The mean emission of fluoride in A2 subcategory at day 15 was 0.261 ± 0.027 ppm. The mean emission of fluoride in A3 subcategory at day 15 was 0.098 ±0.002 ppm. The variations in the values in different subcategory in category A was statistically meaningful (p <0.001) with maximum emission of fluoride ion in subcategory A2 at day 15. It was observed that in subcategory A1 and A3 there was continuous decline in emission of fluoride ion as the days progressed. However there was increase in emission of fluoride in A2 subcategory on moving to day 5 from day 1. However from day 5 onwards decline in fluoride emission was observed in A2 subcategory also. (Table 1)

The mean emission of fluoride ion in B1 subcategory at day 1 was 6.811 ± 0.269 ppm. The mean emission of fluoride in B2 subcategory at day 1 was 0.571 ±0.027 ppm. The mean emission of fluoride in B3 subcategory at day 1 was 0.841 ±0.021 ppm. The variations in the values in different subcategory in category B was statistically meaningful (p <0.001) with maximum emission of fluoride ion in subcategory B1 at day 1. The mean emission of fluoride ion in B1 subcategory at day 5 was 0.891 ±0.021ppm. The mean emission of fluoride in B2 subcategory at day 2 was 0.691 ± 0.027ppm. The mean emission of fluoride in B3 subcategory at day 5 was 0.171 ±0.023ppm. The variations in the values in different subcategory in category B was statistically meaningful (p <0.001) with maximum emission of fluoride ion in subcategory B1 at day 5.The mean emission of fluoride ion in B1 subcategory at day 10 was 0.391 ±0.027 ppm. The mean emission of fluoride in B2 subcategory at day 10 was 0.221 ± 0.027 ppm. The mean emission of fluoride in B3 subcategory at day 15 was 0.131 ±0.021 ppm. The variations in the values in different subcategory in category B was statistically meaningful (p <0.001) with maximum emission of fluoride ion in subcategory B1 at day 10.The mean emission of fluoride ion in B1 subcategory at day 15 was 0.294 ±0.027ppm. The mean emission of fluoride in B2 subcategory at day 15 was 0.181 ±0.021ppm. The mean emission of fluoride in B3 subcategory at day 15 was 0.098± 0.003 ppm. The variations in the values in different subcategory under category B was statistically meaningful (p<0.001) with maximum emission of fluoride ion in subcategory B1 at day 15. It was observed that in subcategory B1 and B3 there was continuous decline in emission of fluoride ion as the days progressed. However there was increase in emission of fluoride in B2 subcategory on moving to day 5 from day 1. However from day 5 onwards decline in fluoride emission was observed in B2 subcategory also (Table 2).

On comparing subcategories A1 and B1 it was observed that values of fluoride emission at day one was lesser in A1 (2.912 ±0.169) as compared to B1 (6.811 ±0.269). It was observed that values of fluoride emission at day five was lesser in A1 (0.671 ±0.027) as compared to B1 (0.891 ±0.021). It was observed that values of fluoride emission at day ten was lesser in A1 (0.271 ±0.021) as compared to B1 (0.391 ±0.027). It was observed that values of fluoride emission at day fifteen was lesser in A1 (0.231 ±0.027) as compared to B1 (0.294 ±0.027).The difference in observations was meaningful statistically (Table 3).

On comparing subcategories A2 and B2 it was observed that values of fluoride emission at day one was lesser in A2 (0.431 ±0.023) as compared to B2 (0.571 ±0.027). It was observed that values of fluoride emission at day five in A2 (0.701 ± 0.027) was comparable to B2 (0.691 ± 0.027) (p=0.361). The difference was statistically insignificant. It was observed that values of fluoride emission at day ten was greater in A2 (0.341 ± 0.024) as compared to B2 (0.221 ± 0.027). It was observed that values of fluoride emission at day fifteen was greater in A2 (0.261 ± 0.027) as compared to B2 (0.181 ±0.021).The difference in observations was meaningful statistically. (Table 4)

On comparing subcategories A3 and B3 it was observed that values of fluoride emission at day one was lesser in A3 (0.441 ±0.024) as compared to B3 (0.841 ±0.021). It was observed that values of fluoride emission at day five in A3 (0.161 ± 0.021) was comparable to B3 (0.171 ±0.023) (p=0.589). The difference in observations was non-meaningful statistically. It was observed that values of fluoride emission at day ten in A3 (0.132 ±0.021) was comparable to B3 (0.131 ±0.021)(p=0.765) It was observed that values of fluoride emission at day fifteen in A3 (0.098 ±0.002) was comparable to B3 (0.098± 0.003)(p=0.368).The difference in observations was non-meaningful statistically. (Table 5)

## Discussion:

GIC is the ideal dental restorative material used in paediatric operational dentistry due to its beneficial properties, including its capacity to cling to dental tissues and emit fluoride for an extended period of time [8, 9]. But there are also a number of disadvantages, such as early moisture susceptibility, poor cosmetics, a lengthy setting time, diminished mechanical strength, and poor good adhesion [10,11]. To protect the tooth restorative material from such mishaps, it is suggested that one should use an enamel surface covering reagent right away [12, 13, 14]. These cover varnishes, all-purpose emollients like petroleum gel, and adhesive resins that cure with light and liquid. The scientific literature hasn't looked into the effects of washing solutions and surface treatments on the emission of fluoride ions through various glass ionomer replacements in great detail. The goal of the current in-vitro experiment was to compare and evaluate the fluoride emission properties of regular GIC, Zirconomer cement, and those materials combined with and without surface coatings. In our study it was observed that in subcategory A1 (GIC with no coating) and A3 (GIC with petroleum coating) there was continuous decline in emission of fluoride ion as the days progressed. However there was increase in emission of fluoride in A2 subcategory (GIC with G-Coat) on moving to day 5 from day 1. However from day 5 onwards decline in fluoride emission was observed in A2 subcategory also.It was observed that in subcategory B1 (Zircomer with no coating) and B3 (zircomer with petroleum covering), there was continuous decline in emission of fluoride ion as the days progressed. However, there was increase in emission of fluoride in B2 (zircomer with G-coat) sub-category on moving to day 5 from day 1. However from day 5 onwards decline in fluoride emission was observed in B2 subcategory also. Similar fluoride emission patterns have been seen in investigations by Yap *et al*. [12], De Moor *et al*. [10], Yip and Smales. [11] Tooth decay is one of the most prevalent chronic diseases that affect humans. In most developed countries, it continues to be a substantial health danger to the majority of adults and school-age children [15, 16]. Tooth decay was previously rare in the majority of underdeveloped countries, but this is no longer the case. Changes in food habits and insufficient exposure to fluoride are partially to blame for this. On the other hand, enhanced public health initiatives, such as the successful use of fluorides, modified lifestyles, and the adoption of innovative restorative materials, have resulted in a decrease in dental cavities in the majority of developed countries during the past 20 years [17, 18, 19].

In this study, while comparing subcategories A2 (GIC with G-coat) and B2 (zircomer with G-coat) it was observed that values of fluoride emission at day one was lesser in A2 (0.431 ±0.023) as compared to B2 (0.571 ±0.027). It was observed that values of fluoride emission at day five in A2 (0.701 ± 0.027) was comparable to B2 (0.691 ± 0.027). (p=0.361). The difference was statistically insignificant. It was observed that values of fluoride emission at day ten was greater in A2 (0.341 ± 0.024) as compared to B2 (0.221 ± 0.027). It was observed that values of fluoride emission at day fifteen was greater in A2 (0.261 ± 0.027) as compared to B2 (0.181 ±0.021).The difference in observations was meaningful statistically. To solve these issues and improve the restoration's mechanical and structural qualities, zirconia fillers are included in GIC's glass component. For people with a high prevalence of cavities, Zirconomer (Zirconia reinforced Glass ionomer), which combines endurance, superior strength, and constant fluoride protection, is the ideal posterior dental restorative material [20, 21, 22]. The GIC, which is water sensitive (hydrophilic) and includes a sizable amount of loosely linked water, is the most well-known material in the research [23, 24, 25]. Due to dehydration and contamination of the substance present in water and saliva, the cement consisting of ions of calcium, aluminium, and silicate chemical agent will be scrubbed out during the initial stages of the chemical reaction for setting of cement. This will result in a loss of lustre, a decline in physical capacity, and an elevated risk of breakdown.

Here, comparing subcategories A3 (GIC covered with petroleum) and B3 (zircomer coated with petroleum it was observed that values of fluoride emission at day one was lesser in A3 (0.441 ±0.024) as compared to B3 (0.841 ±0.021). It was observed that values of fluoride emission at day five in A3 (0.161 ± 0.021) was comparable to B3 (0.171 ±0.023) (p=0.589). The difference in observations was non-meaningful statistically. It was observed that values of fluoride emission at day ten in A3 (0.132 ±0.021) was comparable to B3 (0.131 ±0.021) and (p=0.765). It was observed that values of fluoride emission at day fifteen in A3 (0.098 ±0.002) was comparable to B3 (0.098± 0.003) (p=0.368).The difference in observations was not meaningful statistically. Due to dispersion across cracks and pore spaces, fluoride emission significantly decreased until the day fifteen in both categories. Studies by Mazzaoui *et al*. [19], Castro *et al*. [18] and McKnight-Hanes [17] were congruent with the trend of fluoride emission from surfaces covered subgroups.

## Conclusion:

Both materials studied (GIC and Zirconomer) exhibited fluoride emission whether or not they were surface-coated for protection. Although there were variations among the categories, the trend of fluoride release - first the initial surge followed by steady release-was consistent through course of the research. Zirconomer emitted more fluoride, according to the findings, and is roughly the same as traditional GIC.

## Figures and Tables

**Table 1:** Mean fluoride emission (ppm) for category A compared within groups.

	**Day 1 (Mean ± SD)**	**Day 5 (Mean ± SD**)	**Day 10 (Mean ± SD)**	**Day 15 (Mean ± SD)**
A1	2.912 ±0.169	0.671 ±0.027	0.271 ±0.021	0.231 ±0.027
A2	0.431 ±0.023	0.701 ± 0.027	0.341 ± 0.024	0.261 ± 0.027
A3	0.441 ±0.024	0.161 ± 0.021	0.132 ±0.021	0.098 ±0.002
P value	<0.001	<0.001	<0.001	<0.001
Inference	Significant	Significant	Significant	Significant

**Table 2:** Mean fluoride emission (ppm) for category B compared within category.

	**Day 1 (Mean ± SD)**	**Day 5 (Mean ± SD)**	**Day 10 (Mean ± SD)**	**Day 15 (Mean ± SD)**
B1	6.811 ±0.269	0.891±0.021	0.391±0.027	0.294 ±0.027
B2	0.571±0.027	0.691 ± 0.027	0.221 ± 0.027	0.181±0.021
B3	0.841±0.021	0.171±0.023	0.131±0.021	0.098± 0.003
P value	<0.001	<0.001	<0.001	<0.001
Inference	Significant	Significant	Significant	Significant

**Table 3:** Mean fluoride release across categories for subcategories of A1 and B1.

	**Day 1 (Mean ± SD)**	**Day 5 (Mean ± SD)**	**Day 10 (Mean ± SD)**	**Day 15 (Mean ± SD)**
A1	2.912 ±0.169	0.671 ±0.027	0.271 ±0.021	0.231 ±0.027
B1	6.811 ±0.269	0.891 ±0.021	0.391 ±0.027	0.294 ±0.027
P value	<0.001	<0.001	<0.001	<0.001
Inference	Significant	Significant	Significant	Significant

**Table 4:** Mean fluoride release across categories for subcategories of A2 and B2.

	**Day 1 (Mean ± SD)**	**Day 5 (Mean ± SD)**	**Day 10 (Mean ± SD)**	**Day 15 (Mean ± SD)**
A2	0.431 ±0.023	0.701 ± 0.027	0.341 ± 0.024	0.261 ± 0.027
B2	0.571 ±0.027	0.691 ± 0.027	0.221 ± 0.027	0.181 ±0.021
P value	<0.001	0.361	<0.001	<0.001
Inference	Significant	Insignificant	Significant	Significant

**Table 5:** Mean fluoride release across categories for subcategories of A3 and B3.

	**Day 1 (Mean ±SD)**	**Day 5 (Mean ±SD)**	**Day 10 (Mean ±SD)**	**Day 15 (Mean ±SD)**
A3	0.441 ±0.024	0.161 ±0.021	0.132 ±0.021	0.098 ±0.002
B3	0.841 ±0.021	0.171 ±0.023	0.131 ±0.021	0.098±0.003
P value	<0.001	0.589	0.765	0.368
Inference	Significant	Insignificant	Insignificant	Insignificant
